# Phenotypic plasticity closely linked to climate at origin and resulting in increased mortality under warming and frost stress in a common grass

**DOI:** 10.1002/ece3.4848

**Published:** 2019-01-18

**Authors:** Juergen Kreyling, Sébastien J. Puechmaille, Andrey V. Malyshev, Fernando Valladares

**Affiliations:** ^1^ Institute of Botany and Landscape Ecology, Experimental Plant Ecology Greifswald University Greifswald Germany; ^2^ Zoological Institute and Museum, Applied Zoology and Nature Conservation Greifswald University Greifswald Germany; ^3^ Museo Nacional de Ciencias Naturales CSIC Madrid Spain; ^4^ Present address: ISEM, Univ Montpellier, CNRS, EPHE, IRD Montpellier France

**Keywords:** climate change, inter‐specific variation, intra‐specific variation, local adaptation, phenotypic plasticity, winter ecology

## Abstract

Phenotypic plasticity is important for species responses to global change and species coexistence. Phenotypic plasticity differs among species and traits and changes across environments. Here, we investigated phenotypic plasticity of the widespread grass *Arrhenatherum elatius* in response to winter warming and frost stress by comparing phenotypic plasticity of 11 geographically and environmentally distinct populations of this species to phenotypic plasticity of populations of different species originating from a single environment. The variation in phenotypic plasticity was similar for populations of a single species from different locations compared to populations of functionally and taxonomically diverse species from one environment for the studied traits (leaf biomass production and root integrity after frost) across three indices of phenotypic plasticity (RDPI, PIN, slope of reaction norm). Phenotypic plasticity was not associated with neutral genetic diversity but closely linked to the climate of the populations’ origin. Populations originating from warmer and more variable climates showed higher phenotypic plasticity. This indicates that phenotypic plasticity can itself be considered as a trait subject to local adaptation to climate. Finally, our data emphasize that high phenotypic plasticity is not *per se* positive for adaptation to climate change, as differences in stress responses are resulting in high phenotypic plasticity as expressed by common plasticity indices, which is likely to be related to increased mortality under stress in more plastic populations.

## 1 INTRODUCTION

Phenotypic plasticity, that is, the capacity of a genotype to realize different phenotypic values for a given trait under altered environmental conditions (Valladares, Sanchez‐Gomez, & Zavala, 2006), is of high interest in ecological and evolutionary research for improving the understanding of species coexistence (Turcotte & Levine, 2016) and responses to global change (Merila & Hendry, 2014). It is generally assumed that phenotypic plasticity differs among species and traits and that it changes across environments (Bradshaw, 2006; Pemac & Tucić, 1998; Richards, Bossdorf, Muth, Gurevitch, & Pigliucci, 2006; Valladares, Balaguer, Martinez‐Ferri, Perez‐Corona, & Manrique, 2002; West‐Eberhard, 2003). Here, we directly compared the hetero‐ and conspecific variation in phenotypic plasticity, explored the relative influence of environment and genetic diversity on phenotypic plasticity, and linked phenotypic plasticity to mortality under stress.

Based on morphological and physiological differences, one would expect a higher variation in phenotypic plasticity among heterospecific populations rather than among conspecific populations. However, this expectation may depend on environmental conditions and traits considered (Schlichting & Smith, 2002). Traits under selective pressure and related to stress response may actually be as variable within a given species, that is, for populations originating from different environments, as between species if the populations for all species stem from the same environment (Des Roches et al., 2018; Malyshev et al., 2016; Poirier, Durand, & Volaire, 2012).

Environmental conditions should affect phenotypic plasticity (van Kleunen & Fischer, 2005) with phenotypic plasticity expected to increase in response to more variable conditions over time (Ghalambor, Huey, Martin, Tewksbury, & Wang, 2006; Lázaro‐Nogal et al., 2015; Molina‐Montenegro & Naya, 2012). On the other hand, genetic diversity should also affect phenotypic plasticity (Hughes, Inouye, Johnson, Underwood, & Vellend, 2008). Genetic diversity represents the basis for the expression of specific reaction norms of morphological traits (Nicotra et al., 2010; Scheiner, 1993). The final phenotype is then determined by epigenetic, transcriptional, or posttranscriptional regulation depending on environmental conditions (Nicotra et al., 2010; Zhang, Fischer, Colot, & Bossdorf, 2013). As a result of this, some studies suggest that even neutral genetic diversity facilitates phenotypic plasticity (Doi, Takahashi, & Katano, 2010; Ehlers, Worm, & Reusch, 2008; Harter et al., 2015; Hughes et al., 2008; Jump, Marchant, & Peñuelas, 2009), while others report high phenotypic plasticity combined with low genetic diversity (Arnaud‐Haond, Marbà, Diaz‐Almela, Serrão, & Duarte, 2010; Frenot et al., 1999; Geng et al., 2006; Khankhet et al., 2014). In light of anthropogenic climate change and loss of biodiversity, the effects of climatic parameters and genetic diversity are of ecological importance but have been rarely compared directly to assess their influence on phenotypic plasticity.

Phenotypic plasticity is commonly assumed to have positive implications for species facing environmental change (Richter et al., 2012; Scheiner, 1993; Schlichting, 1986; West‐Eberhard, 2003; Yeh & Price, 2004). Concerning adaptation to changing environmental conditions, phenotypic plasticity may either buy time for adaptation (Chevin, Lande, & Mace, 2010) or even directly facilitate adaptation (Ghalambor, McKay, Caroll, & Reznick, 2007). However, phenotypic plasticity comes with costs (DeWitt, Sih, & Wilson, 1998) and may be adaptive, maladaptive, or neutral with regard to an individual's fitness, that is, phenotypic plasticity can also prevent adaptation under certain conditions (Ghalambor et al., 2007). Maladaptive or nonadaptive plastic changes might occur as a result of stress, and evidence from some case studies also suggests reduced performance with increasing phenotypic plasticity (Gotthard, Nylin, & Nylin, 1995; Grether, 2005; Merila & Hendry, 2014; Michalski, Malyshev, & Kreyling, 2017; Teplitsky, Mills, Alho, Yarrall, & Merila, 2008).

Here, we investigated phenotypic plasticity of the common and widespread grass *Arrhenatherum elatius* to winter warming and frost stress. We compared variation in phenotypic plasticity of leaf biomass production and root integrity of 11 environmentally distinct populations of this species to variation in phenotypic plasticity among 8 species whose populations all stem from one environment. Previous studies on *A. elatius* found high phenotypic and genetic variability for quantitative traits (Mahmoud, Grime, & Furness, 1975; Petit & Thompson, 1998) and differentiation across spatial scales (Kreyling et al., 2012; Petit & Thompson, 1998). We expand these studies by linking phenotypic plasticity to genetic diversity and to climatic origin of the populations. We further evaluated the relationship between phenotypic plasticity and adaptation to environmental stress by linking phenotypic plasticity to mortality. We hypothesized that (a) conspecific variation in phenotypic plasticity (across populations originating from different locations) is similarly high as heterospecific variation in phenotypic plasticity (different species from one location) for stress‐related traits presumably under selection, that is, leaf biomass production and root integrity after frost stress. We further expected that (b) differences in phenotypic plasticity among populations of a single species are related both to climate at their origin and to their neutral genetic diversity. Finally, we hypothesized, given that plasticity has costs, (DeWitt et al., 1998) that (c) the most plastic populations have the highest mortality under stress.

## 2 MATERIALS AND METHODS

To analyze phenotypic plasticity within and among species and link it to performance, climate at origin, and genetic diversity, we used data from a winter warming plus frost experiment (see Malyshev et al., 2016 for details) on four grasses, two nonleguminous forbs, two leguminous forbs (all from one geographic origin), and 11 populations of the common and widespread grass *Arrhenatherum elatius* (L.) P.Beauv. ex J.Presl & C.Presl from different European populations (Ireland, Spain, Germany and Poland). *A. elatius* is native in and widely distributed throughout Europe and introduced to North America, New Zealand, and Australia. Populations of *A. elatius* used in the experiment were chosen as genetically distinct seed lines (acquired from the seed bank at the Leibniz Institute of Plant Genetics and Crop Plant Research) based on previous genetic analyses which had been carried out on the same seed sources (Michalski et al., 2010). Within‐species variation was represented by 11 genetically distinct populations from across Europe (Table 1). For this species, there is evidence of local adaptation in biomass production after spring frost at the continental scale, whereby populations originating from regions with a higher incidence of spring frost events are more resilient to spring frost damage (Kreyling et al., 2012). Among‐species variation was represented by four grasses (*Festuca pratensis* Huds., *Holcus lanatus* L., *Alopecurus pratensis* L., *Arrhenatherum elatius*), two nonleguminous forbs (*Geranium pratense* L., *Plantago lanceolata* L.) and two leguminous forbs (*Lotus corniculatus* L., *Trifolium pratense* L.); all sharing the same seed origin (see Table 1). Among‐species variation therefore covered a broad gradient of functionally and taxonomically distinct species.

**Table 1:** Geographic, climatic, and genetic information about the used populations of *Arrhenatherum elatius* forming the within‐species group (upper part) and the species forming the among‐species group (lower part). Climatic data from WorldClim (Hijmans et al., 2005) with MAT being the mean annual temperature and MAP the mean annual precipitation. Neutral genetic diversity of ecotypes was quantified by the proportion of polymorphic loci and by the mean pairwise Jaccard dissimilarity among individuals within populations (*J*), based on amplified length polymorphism (AFLP; see Michalski et al., 2010 for details). Note that PL.A and PL.B originate from Germany but are genetically more similar to polish populations

Species	Abbreviation	Latitude (°N)	Longitude (°E)	Elevation (m a.s.l.)	MAT (°C)	MAP (mm)	Polymorphic Loci (%)	*J*
*Arrhenatherum elatius*	ES.A	43.255	−7.289	600	12.0	1,010	73.1	0.389
	ES.B	42.628	−8.118	545	12.4	1,330	79.6	0.372
	ES.C	43.233	−8.016	280	12.4	1,207	71.5	0.343
	IE.A	52.645	−8.954	12	10.3	1,012	75.8	0.396
	IE.B	53.515	−8.851	42	9.3	1,079	83.9	0.405
	IE.C	52.046	−9.511	25	10.2	1,324	74.2	0.353
	DE.B	51.748	10.753	470	6.8	828	69.9	0.317
	DE.C	51.893	12.024	60	9.2	489	72.0	0.317
	PL.A	50.548	10.787	450	7.0	705	84.9	0.433
	PL.B	51.642	10.924	490	6.9	762	73.1	0.330
	PL.C	50.570	21.680	490	8.1	561	81.2	0.353
	Ae	50.610	10.700	455	6.6	764	73.7	0.341
*Holcus lanatus*	Hl	49.167	9.567	460	9.6	676		
*Alopecurus pratensis*	Ap	49.167	9.567	460	9.6	676		
*Geranium pratense*	Gp	49.167	9.567	460	9.6	676		
*Plantago lanceolata*	Pl	49.167	9.567	460	9.6	676		
*Lotus corniculatus*	Lc	49.167	9.567	460	9.6	676		
*Trifolium pratense*	Tp	49.167	9.567	460	9.6	676		

The seeds stemmed from the seed bank at the Leibniz Institute of Plant Genetics and Crop Plant Research (*Arrhenatherum* populations) and from a commercial seed supplier (Rieger‐Hofmann GmbH; all other species). Plants were cultivated from seed, and the seedlings were then transplanted into plastic pots (5 cm diameter × 7 cm), using seed compost soil (Einheitserde Classic, Germany). NPK (Mg) liquid fertilizer (15 + 10 + 15 (+2)) was applied once at the start of the experiment at a concentration of 1 g/L (Hakaphos Blau, COMPO EXPERT, Germany). During October and November, the plants were grown in a greenhouse at the Leibniz Institute of Plant Genetics and Crop Plant Research, where night and daytime temperatures averaged 6.4°C and 20.0°C, respectively. Light was provided with 400‐W lamps (approximately 600 µmol m^−2^ s^−1^), with a 10 hr photoperiod. Plants were transferred to climate chambers at the University of Bayreuth at the end of November and for two weeks; the day and night time temperatures were lowered to 10°C and 6°C, respectively, photoperiod was decreased to 9 hr, and PAR light intensity was 200 µmol m^−2^ s^−1^. To complete plant cold acclimation, the photoperiod was lowered to 8 hr for one month, with soil surface temperature averaging 0.0°C (minimum −6.2°C; maximum +5.8°C). Plants were kept at −1.5°C prior to thaw treatments, which took place on 12–23 February 2012.

The thaw and frost manipulation was designed based on the fact that snow cover in cool temperate sites decreases with climate change (Kreyling & Henry, 2011), leading to reduced insulation and exposing overwintering herbaceous plants to more variable temperature regimes (Kreyling, 2010). More frequent warm spells can trigger deacclimation of cold‐acclimated plants within hours of warming, leaving plants susceptible to frost damage when freezing temperatures return (Bokhorst, Bjerke, Tømmervik, Callaghan, & Phoenix, 2009; Kalberer, Wisniewski, & Arora, 2006). Intensity and duration of frost events may not decrease within this century, despite the global warming reducing their frequency of occurrence (Kodra, Steinhaeuser, & Ganguly, 2011). In that light, a warming and frost experiment was conducted on the hardened plants. On 12 February, all plants (10 plants per population and species per treatment) were assigned to one of three thaw treatments: 12 hr at 4°C (control), 2 days at 9°C, or 6 days at 9°C (warming). We focused on the length of thaw on frost tolerance and not on the effect of minimum temperature itself. Freeze–thaw events are known for their ecological importance, and the control therefore included the same number of freeze–thaw events as the manipulations, while the thawing periods differed in length and maximum temperature. Potential changes in frost tolerance due to the respective thaw periods were assessed by quantifying the responses of the plants to a severe frost event. Frost was administered for 24 hr right after the warm spell manipulations. The treatments and plants were switched between three climate chambers every second day, and the environmental conditions were closely monitored throughout the experiment. Minimum chamber temperatures in the control and warming treatments reached −11.9°C and −8.7°C, respectively, while the respective mean temperatures were −7.2°C and −6.7°C. The lower minimum temperature in the control treatment still resulted in plants having more than doubled the growth performance than plants which had experienced milder frost, but after the prolonged warming. After thawing, all plants were repotted (8 cm × 8 cm × 20 cm deep pots) and transferred to a greenhouse. Temperature was increased by 2°C every 10 d to simulate spring, reaching ~14°C on 14 March.

### 2.1 Response parameters

Above‐ground biomass was harvested one month after the frost, with brown or discolored tissue assigned as dead tissue and separated from green tissue (further used as leaf biomass). Leaf biomass was dried to a constant weight at 60°C and weighed. Here, leaf biomass is not just a measure of performance but rather a quantification of a plant`s strategy to cope with the eminent trade‐off between an early start of growth and risking frost damage of deacclimated tissue.

A subset of plants was used for destructive analysis of root integrity (*n* = 4 per population and species). Root functional integrity was assessed immediately after the frost treatment by measuring ^15^N uptake. Plants and soil were first transferred into plastic cups (5 cm diameter × 10 cm deep). Twelve mL of 100 µM ^15^NH_3_^15^NO_3_ solution was injected 1.5 cm deep into the soil in three aliquots, equidistant from the center. After 22 hr of incubation at 20°C, the plants were rinsed free of soil, washed with 50 ml of 5 mM KCl and 0.5 mM CaCl_2_, then rinsed with 200 ml of deionized water to remove ammonium passively adsorbed in the root cell walls via cation exchange (Epstein, Schmid, & Rains, 1963). Roots were excised, oven‐dried at 60°C for 48 hr, fine‐milled, and analyzed using mass spectroscopy analysis at the laboratory of Isotope Biogeochemistry, BayCEER, University of Bayreuth, with a combination of an elemental analyzer (Carlo Erba NC 2500, CE Instruments, Italy) and an isotope mass spectrometer (Delta Plus, Thermo Fisher Scientific, Germany).

### 2.2 Plasticity indices

Phenotypic plasticity for each population or species was quantified by three different indices according to Valladares et al. (2006). (a) Relative Distances Plasticity Index (RDPI = Absolute phenotypic distances between individuals of same group and different environments, divided by the bigger of the two phenotypic values for two environments; that is, control and 6 days thaw treatment), (b) Phenotypic Inertia (PIN = (Σ(Survival_i_ × performance_i_))/(*n* × *SD*) for two environments; that is, control and 6 days thaw treatment), and (c) slope of reaction norm with hourly temperature sums during the thaw treatment as quantitative difference among the environments for all three environments. Results for all three indices calculated via custom R (version 3.4.0; R Core Team, 2017) scripts were qualitatively similar and can be found in the electronic appendix (see Table S1 and Figure S1). For the main text, only RDPI is presented for its positive performance in previous tests (Valladares et al., 2006).

### 2.3 Climate and genetic diversity

Climatic information for the locations of origin of the populations was taken from bioclimatic variables downloaded from WorldClim (Hijmans, Cameron, Parra, Jones, & Jarvis, 2005). We tested mean annual temperature, mean temperature of warmest quarter, mean temperature of coldest quarter, annual precipitation, variance in monthly precipitation (CV), and precipitation of warmest quarter for relations with phenotypic plasticity. Genetic diversity of the populations was based on amplified length fragment polymorphisms (AFLP, Michalski et al., 2010) and expressed as the proportion of polymorphic loci and the mean pairwise Jaccard distance between all individuals of one population (*J*) (see Michalski et al., 2010 for details). *Arrhenatherum elatius* is an autotetraploid species with complex meiosis. Therefore, a strictly simple band‐based approach was taken with Jaccard distance to overcome potential bias by homoplasy, as this index considers only band presence as information.

### 2.4 Statistical analysis

Levene's tests were applied to test for equality of variances in phenotypic plasticity among and within species. Ordinary least‐squares regression was conducted to test for univariate relationships between phenotypic plasticity and the climatic and genetic variables. A variance partitioning based on linear regression and redundancy analysis ordination (RDA; Legendre, 2008) was used to differentiate between the percentages of variance in phenotypic plasticity that were explained by genetic diversity and climate of origin jointly and individually, using the two parameters of genetic diversity and the six climate variables described above. Finally, we linked phenotypic plasticity to mortality in the 6 days thawing treatment by ordinary least‐squares regression. All tests were run in R (version 3.4.0; R Core Team, 2017).

## 3 RESULTS

**Figure 1:**
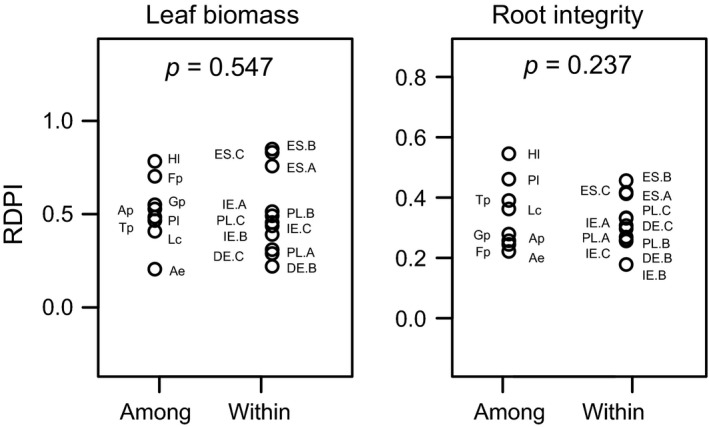
Comparison of phenotypic plasticity (RDPI) in leaf biomass and root integrity after winter warming and frost stress among 8 species from a common origin and within one species (*Arrhenatherum elatius*) across 11 populations from different origins. See Table 1 for further information on species and populations

The variation in phenotypic plasticity within one species (RDPI ranging from 0.22 to 0.86 for leaf biomass and 0.18–0.46 for root integrity for 11 *A. elatius* populations from different locations) did not differ from the range among species (RDPI 0.21–0.79 for leaf biomass and 0.22–0.55 for root integrity for 8 species from one location; Figure 1; Levene test for both traits and all three plasticity indices: *p* > 0.2). Absolute RDPI values showed comparably low values (<0.3) for some species originating from the same region (“among”), for example, *A. elatius* or *L. corniculatus* and for some populations within a species (“within”), for example, IE.B or DE.B. Relatively high RDPI values (>0.7) occurred also both among (e.g., *H. lanatus* and *F. pratensis*) and within species (e.g., ES.B and ES.C). For the latter group, the Spanish populations appeared to be more plastic while no clear pattern among functional groups (grasses, forbs, legumes) was observed among species (Figure 1).

The differences in phenotypic plasticity among populations of *A. elatius* were not linked to neutral genetic diversity (Table 2). Tight correlations between phenotypic plasticity and climatic parameters were observed, with strong positive correlations between mean annual temperature and phenotypic plasticity of leaf biomass but also between mean summer temperature and phenotypic plasticity of root integrity (Table 2). However, all tested climate parameters showed significant relations to phenotypic plasticity of at least one plant trait. Generally, phenotypic plasticity in our experiment increased with warmer mean temperatures at the origin of the studied populations. A more complex picture was observed concerning precipitation with phenotypic plasticity being positively linked to annual precipitation, but also to temporal variance in precipitation while it was negatively linked to summer precipitation. Variance partitioning between climate and genetic diversity revealed that phenotypic plasticity was mainly influenced by climate (Table 3). Genetic diversity had little effects, and no jointly explained variance (climate and genetic diversity) was detected (Table 3).

**Table 2:** Corrected *R* ^2^ of linear regression between plasticity of populations (RDPI) and genetic diversity and climate of origin for 11 populations of *Arrhenatherum elatius*

Realm	Parameter	Leaf biomass	Root integrity
Genetic	Proportion of polymorphic loci	0.00	0.00
	Mean pairwise Jaccard dissimilarity	0.00	0.00
Climatic	Mean annual temperature	0.73***	0.50**
	Mean temperature of warmest quarter	0.33*	0.70***
	Mean temperature of coldest quarter	0.53**	0.18
	Annual precipitation	0.34*	0.01
	Variance in precipitation (CV)	0.57**	0.55**
	Precipitation of warmest quarter	0.41*	0.64**

*** *p* < 0.001,

** *p* < 0.01,

* *p* < 0.05.

**Table 3:** Explained variance in phenotypic plasticity (RDPI) by climate (mean annual temperature, mean temperature of warmest quarter, mean temperature of coldest quarter, annual precipitation, variance in precipitation (CV), precipitation of warmest quarter) and genetic diversity (proportion of polymorphic loci, mean pairwise Jaccard dissimilarity) as analyzed by variance partitioning. Data for 11 populations of *Arrhenatherum elatius*

Explanatory variable	Leaf biomass (%)	Root integrity (%)
Climate	84	92
Genetic diversity	6	2
Genetic diversity and climate jointly	0	0

**Figure 2:**
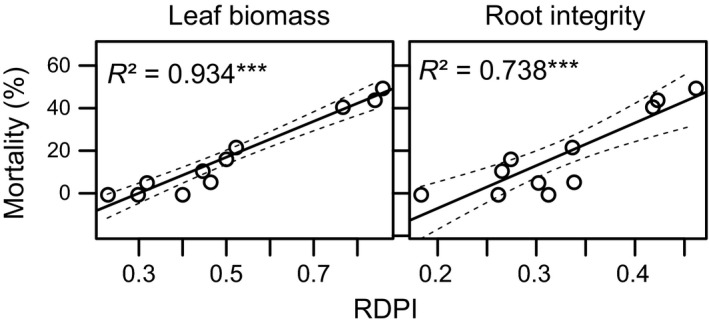
Mortality of *Arrhenatherum elatius* populations versus phenotypic plasticity (RDPI) in leaf biomass production and root integrity after the warming and frost treatment. Solid lines show the results of linear regressions with corrected *R* ^2^ being reported and dotted lines show the 95% confidence interval. ***p < 0.001

Mortality due to the warming and frost treatment varied between 0% and 50% among the populations of *A. elatius*. More plastic populations showed higher mortality as indicated by tight positive correlations between mortality and phenotypic plasticity (Figure 2). Among species, mortality ranged between 0% (*F. pratensis*) and 40% (*H. lanatus*) without a significant correlation with phenotypic plasticity (*p* > 0.2 for all combinations of traits and plasticity indices).

## 4 DISCUSSION

Phenotypic plasticity of populations in two stress‐related traits varied as much for 11 populations from different origins within one species as among functionally and taxonomically diverse species all originating from the same environment. We assume that this is a common feature for stress‐related traits under selective pressure as the environment selects for trait convergence (Des Roches et al., 2018; Malyshev et al., 2016; Poirier et al., 2012). Accordingly, among‐species plasticity commonly exceeds within‐species plasticity in traits presumably not under selection such as leaf morphological traits or nutrient stoichiometry (Albert et al., 2010; Kichenin et al., 2013).

Our data show high within‐species variation in phenotypic plasticity for one species. An important question, of course, would be whether all species contain populations varying strongly in phenotypic plasticity. To our knowledge, no such data are available for many populations of many species. If exist, it would allow to test whether there are common patterns describing how environmental parameters drive phenotypic plasticity across species and to identify environmental parameters selecting for low or high phenotypic plasticity. Our study (Table 2) generally supports the notion that phenotypic plasticity increases in response to temporally more variable environmental conditions (Ghalambor et al., 2006; Lázaro‐Nogal et al., 2015; Molina‐Montenegro & Naya, 2012).

Phenotypic plasticity was not related to neutral genetic diversity in our study. Some studies suggest that neutral genetic diversity facilitates phenotypic plasticity (Doi et al., 2010; Ehlers et al., 2008; Harter et al., 2015; Hughes et al., 2008; Jump et al., 2009) because genetic diversity within a population increases the possibility of possessing alleles or allele combinations that are advantageous in terms of response capability to environmental change (Jump et al., 2009; Nicotra et al., 2010). However, other studies report high phenotypic plasticity combined with low neutral genetic diversity (Arnaud‐Haond et al., 2010; Frenot et al., 1999; Geng et al., 2006). Of course, neutral genetic diversity is not necessarily correlated with adaptive genetic diversity and fixation of favorable alleles, which could be linked to phenotypic plasticity, depends on effective population size. The key for a better understanding of phenotypic plasticity is to know what determines phenotypic plasticity mechanistically—is it genetically encoded or largely influenced by the environment; and what role epigenetics play in phenotypic plasticity. Our direct comparison showed that, at least for our study system, climate at origin was more strongly linked to stress‐related phenotypic plasticity than neutral genetic diversity.

Phenotypic plasticity is commonly assumed to be beneficial for species facing environmental change (Scheiner, 1993; Schlichting, 1986; West‐Eberhard, 2003; Yeh & Price, 2004). This notion might often be correct, but costs of phenotypic plasticity (DeWitt et al., 1998) and the fact that phenotypic plasticity may be adaptive, maladaptive, or neutral with regard to an individual's fitness (Ghalambor et al., 2007) should always be kept in mind. Maladaptive or nonadaptive plastic changes might occur as a result of stress (Gotthard et al., 1995; Grether, 2005; Merila & Hendry, 2014; Michalski et al., 2017; Teplitsky et al., 2008). Our case study provides clear evidence for maladaptive phenotypic plasticity as mortality increased with phenotypic plasticity, that is, increased variation in leaf biomass production and root integrity was largely caused by an increased mortality in response to winter warming and frost (Figure 2). This outcome, however, is inherent to the nature of the indices of phenotypic plasticity; indices of phenotypic plasticity increase as individuals of the same population increasingly differ in their response to different treatments. Consequently, phenotypic plasticity will be maximal for stress‐related traits if some individuals do well and others very poorly, eventually dying. Interestingly, the same pattern was found for a plasticity index explicitly taking mortality into account (PIN) and for plasticity values not taking mortality into account (RDPI, slope of reaction norm; see Supporting Information Figure S2). While phenotypic plasticity in stress‐related traits is of high ecological importance, high phenotypic plasticity in such traits quantified by common plasticity indices should therefore not be misunderstood as high potential to persist and/or to adapt to environmental change.

Genetic impoverishment and increased drift toward the north have been described for *A. elatius* (Michalski et al., 2017). Selecting the more diverse linages from the south for assisted migration (Kreyling et al., 2011; Vitt, Havens, Kramer, Sollenberger, & Yates, 2010) as management action in face of climate change has been suggested to improve adaptive potential (Michalski et al., 2017). Phenotypic plasticity, however, also needs to be considered and may increase mortality of transplantations if maladaptive. Assisted migration of genetically more diverse, southern populations therefore cannot be generally advised. Phenotypic plasticity was higher in populations from generally warmer origins. This pattern can potentially be explained by a smaller selective pressure for conservative behavior with regard to the trade‐off between dehardening (risk of frost damage) and later onset of growth (reduced biomass production) as compared to colder origins. This selective pressure appears stronger at colder origins, resulting in converged trait variation and, consequently, decreased plasticity in populations stemming from colder climates.

Improved understanding of phenotypic plasticity and its drivers within species is crucial for better modeling species distributions. Most approaches up to now consider species as uniform units without accounting for phenotypic plasticity and differences in phenotypic plasticity among populations (e.g., Thomas et al., 2004; Thuiller, Lavorel, Araujo, Sykes, & Prentice, 2005). Taking population‐specific adaptations and phenotypic plasticity into consideration, however, can affect model outputs considerably (Bush et al., 2016; Oney, Reineking, O'Neill, & Kreyling, 2013; Valladares et al., 2014). Data on phenotypic plasticity among populations that could be used in species distribution modeling, however, are largely missing.

Taken together, our data show that phenotypic plasticity in stress‐related traits of populations from different locations within a single species can vary as much as phenotypic plasticity among species, all originating from the same environment. In our case study, phenotypic plasticity was not linked to neutral genetic diversity but strongly linked to the climate of origin of the populations. This indicates local adaptation to climate for phenotypic plasticity itself. Finally, our data emphasize the notion that high values of phenotypic plasticity, as measured by common metrics, are not per se beneficial for adaptation to climate change, as plasticity in stress responses can be associated with increased mortality in more plastic populations.

## CONFLICT OF INTEREST

None declared.

## AUTHOR CONTRIBUTIONS

All authors conceived the ideas at a RESPONSE summer school; AVM and SJP assembled the data and calculated the indices of PP; JK analyzed the data and wrote the paper; all authors contributed substantially to the text and gave final approval for publication.

## Data Availability

The data supporting our results will be deposited in Dryad upon acceptance of the paper.
