# Supplementary material for: Phenotypic plasticity closely linked to climate at origin and resulting in increased mortality under warming and frost stress in a common grass
Source: Ecol Evol. 2019 Jan 18;9(3):1344–52. doi: 10.1002/ece3.4848 (PMC6374657; doi:10.1002/ece3.4848)
Supplement: Supplementary file 1 [file ECE3-9-1344-s001.docx]

**Supporting Information**


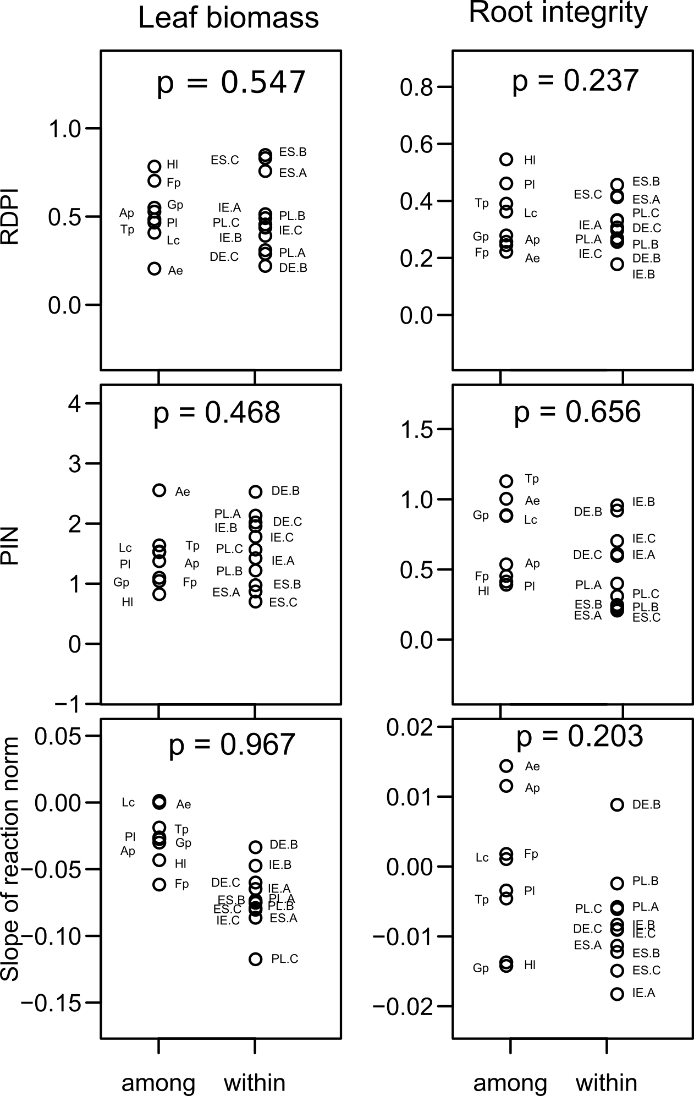


Figure S1: Comparison of phenotypic plasticity (RDPI, PIN and slope of reaction norm) in leaf biomass production and root integrity after winter warming and frost stress among species from a common origin and within one species (*Arrhenatherum elatius*) across 11 populations originating from different origins. See Table 1 for abbreviations of species and populations. P-values according to Levene’s tests for homoscedasticity.

Table S1: Corrected R² of linear regressions between three indices of phenotypic plasticity (RDPI, PIN, slope of reaction norm) of populations versus genetics (Michalski *et al.* 2011) and climate of origin (from worldclim, Hijmans *et al.* 2005) for 11 populations of *Arrhenatherum elatius*.

|  | Parameter | RDPI | | PIN | | Slope of reaction norm | |
| --- | --- | --- | --- | --- | --- | --- | --- |
|  |  | Leaf bio-mass | root inte-grity | Leaf bio-mass | root integrity | Leaf bio-mass | root inte-grity |
| Genetic | Proportion of polymorphic loci | 0.00 | 0.00 | 0.00 | 0.00 | 0.00 | 0.00 |
|  | Mean pairwise Jaccard dissimilarity | 0.00 | 0.00 | 0.00 | 0.00 | 0.00 | 0.09 |
| Climatic | Mean annual temperature | 0.73*** | 0.50** | 0.57** | 0.14 | 0.07 | 0.56** |
|  | Mean temperature of warmest quarter | 0.33* | 0.70*** | 0.29* | 0.43* | 0.18 | 0.09 |
|  | Mean temperature of coldest quarter | 0.53** | 0.18 | 0.38* | 0.00 | 0.00 | 0.46** |
|  | Annual precipitation | 0.34* | 0.01 | 0.15 | 0.00 | 0.00 | 0.09 |
|  | Variance in precipitation (CV) | 0.57** | 0.55** | 0.47** | 0.32* | 0.42* | 0.30* |
|  | Precipitation of warmest quarter | 0.41* | 0.64** | 0.32* | 0.29* | 0.00 | 0.12 |

*** p < 0.001, ** p < 0.01, * p < 0.05


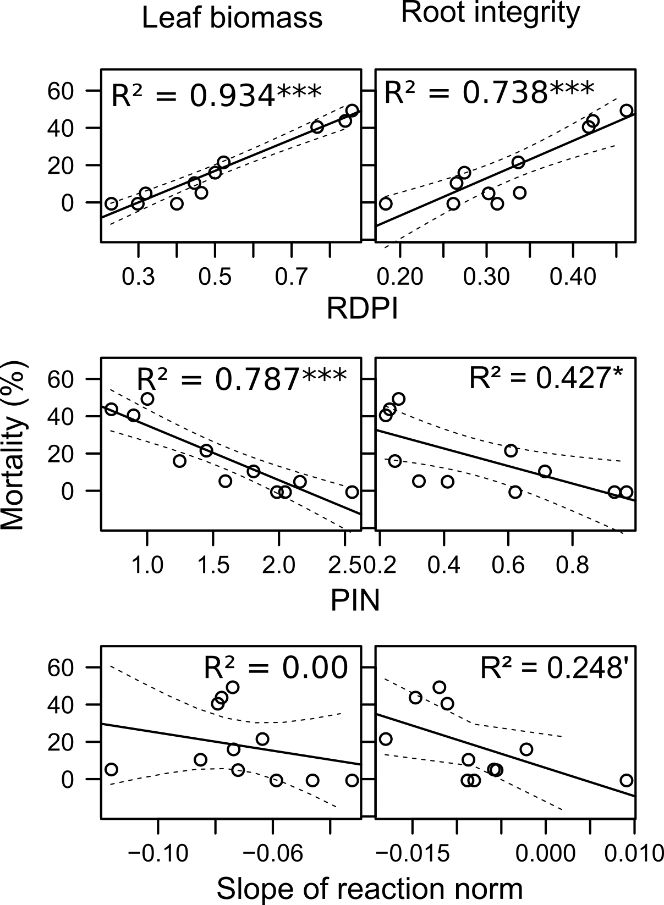


Figure S2: Mortality of 11 *Arrhenatherum elatius* populations versus three indices of phenotypic plasticity (RDPI, PIN, slope of reaction norm) in leaf biomass production and root integrity after the warming and frost treatment. Solid lines show the results of linear regressions with corrected R² being reported and dotted lines show the 95% confidence interval. *** p < 0.001, ** p < 0.01, * p < 0.05, ‘ p < 0.1
